# As an inhibitor of norepinephrine release, dexmedetomidine provides no improvement on stroke-associated pneumonia in mice

**DOI:** 10.3389/fphar.2023.1203646

**Published:** 2023-08-02

**Authors:** Miaomiao Zhou, Qiong Luo, Younian Xu

**Affiliations:** ^1^ Anesthesiology Department, Zhongnan Hospital of Wuhan University, Wuhan, China; ^2^ Anesthesiology Department, Union Hospital, Tongji Medical College, Huazhong University of Science and Technology, Wuhan, China; ^3^ Institute of Anesthesia and Critical Care Medicine, Union Hospital, Tongji Medical College, Huazhong University of Science and Technology, Wuhan, China

**Keywords:** ischemic stroke, stroke-associated pneumonia, dexmedetomidine, norepinephrine, immunity

## Abstract

**Background:** Dexmedetomidine (DEX) is commonly employed as a sedative agent to attenuate sympathetic tone and reduce norepinephrine (NE) levels. In the context of stroke-associated pneumonia (SAP), which is believed to arise from heightened sympathetic nervous system activity and elevated NE release, the precise influence of DEX remains uncertain.

**Methods:** In this study, we generated an SAP model using middle cerebral artery occlusion (MCAO) and examined NE levels, immunological statuses in the brain and periphery, pneumonia symptoms, and extent of infarction. We aimed to determine the effects of DEX on SAP and explore the underlying. Despite its potential to reduce NE levels, DEX did not alleviate SAP symptoms or decrease the infarct area. Interestingly, DEX led to an increase in spleen size and spleen index. Furthermore, we observed a decrease in the CD3+ T cell population in both the blood and brain, but an increase in the spleen following DEX administration. The precise mechanism linking decreased CD3^+^ T cells and DEX’s role in SAP requires further investigation.

**Conclusion:** The clinical use of DEX in stroke patients should be approached with caution, considering its inability to alleviate SAP symptoms and reduce the infarct area. Further research is necessary to fully understand the relationship between decreased CD3^+^ T cells and DEX’s influence on SAP.

## Introduction

Stroke is a leading cause of death worldwide, whose outcome depends on the occurrence of complications (Langhore et al., 2000; Weimar et al., 2002). Stroke-induced immunodepression (SID) can increase susceptibility to infections (Bellinger et al., 2008), among which SAP is the most frequent infectious complication, reportedly occurring in 6.7%–36.98% of stroke patients (Badve et al., 2019). SAP may lead to lengthy hospitalization, poor functional outcomes, and high mortality (Saposnik et al., 2008; Finlayson et al., 2011; Koennecke et al., 2011).

Following a large ischemic stroke, the sympathetic nervous system (SNS) is activated, causing the release of norepinephrine (NE), which subsequently leads to the activation of β2-adrenergic receptors (β2-ARs) (Prass et al., 2006). The β2-ARs, densely expressed on all significant immune cell subtypes, then communicated the signaling pathway and managed the peripheral immune system to be suppressive, by lowering the synthesis and release of inflammatory mediators (Bosmann et al., 2012; Martín-Cordero et al., 2013) and triggering the release of anti-inflammatory cytokines (Hervé et al., 2017; Aaç et al., 2018) from activated macrophages and lymphocytes. Such anti-inflammatory responses are regarded as a compensatory mechanism to prevent the post-ischemic brain from severe and harmful inflammatory responses (Chamorro et al., 2007; Iadecola and Anrather, 2011). However, anti-inflammatory reactions increase susceptibility to systemic infections after stroke, especially pneumonia. A bystander autoimmune factor directed against antigens of the central nervous system can be released as a result of the inflammation caused by pneumonia, which can worsen the prognosis for stroke patients. Therefore, it is critical to prevent stroke-associated pneumonia (Winklewski et al., 2014).

Dexmedetomidine (DEX) is an efficient and highly selective agonist of α2 adrenergic receptors (α2-ARs). By activating presynaptic α2-ARs, DEX reduces sympathetic nerve activity by preventing NE release from the locus coeruleus nucleus (Jorm and Stamford, 1993). Due to its ability to prevent NE release, DEX possesses immune-protective qualities (Wang et al., 2019). DEX protects the brain by preventing microglia from activating, lowering the neuroinflammatory response, and minimizing neuron necrosis and apoptosis, according to both *in vivo* and *in vitro* studies (Kim et al., 2017; Gao et al., 2019). Regarding lung inflammation, researchers have found that DEX reduces inflammatory responses in the lung tissues through a variety of anti-inflammatory channels, including the cholinergic anti-inflammatory system and the TLR4/NF-κB pathway (Wu et al., 2013; Liu et al., 2016).

The present study was undertaken to determine the effects of DEX on cerebral and peripheral immune states in stroke mice and to explore whether DEX would improve symptoms of SAP as well as benefit neuronal outcomes.

## Methods

### Mice/animals

In all experiments, male C57BL/6 mice weighing 22–27 g at 8–10 weeks old were used. Treatment and surgery outcomes were distributed to animals across cages in a random sequence. The Institutional Animal Care and Use Committee of Tongji Medical College, Huazhong University of Science and Technology, gave its approval to all procedures (IACUC Number: 2,419). The National Institutes of Health Guidelines for the Care and Use of Laboratory Animals were followed in all investigations. We attempted to use as few animals as possible.

### SAP model and drug treatment

In our earlier article (Xu et al., 2022), we have shown that the MCAO model in C57/bl6 mice is the ideal model for studying SAP, and the success rate in creating the SAP model was 100%. The exclusion criteria should be: 1. No obvious cerebral infarction was seen by TTC; 2. Mice that dead after the MCAO procedure. The middle cerebral artery occlusion (MCAO) was primarily conducted (Chiang et al., 2011). The agonist of the α2-ARs, dexmedetomidine (DEX) (Hengrui, China), was diluted in 0.9% sodium chloride at 15 mM (25 μg/kg) and then delivered intraperitoneally at P1 (three times daily, every 2 h since 24 h after reperfusion) and P2 (once daily) hours following MCAO (Groups designated as either M-DEX or M-saline, respectively).

### Blood and tissue sample collection

72 h after MCAO, mice were quickly put to death by cervical dislocation. Eyeball removal was used to extract blood, and brain, lung, and spleen tissues were also harvested. The spleen index was calculated as the weight of the spleen (mg)/body weight g)×10.

### Evaluation of sympathetic activity

NE concentrations could be used as a proximate indicator of sympathetic activity (Myers et al., 1981; Gendron et al., 2002; Bieber et al., 2017). To determine the baseline NE level, we treated mice with pentobarbital 50 mg/kg, i.p., to obtain sedation. To prevent NE spillover, we only collected the first two drops of blood after immediately removing the eyeball. Homogenized tissues must first be used for the spleen NE level assessment. Then, centrifuging tissue and blood samples and measuring the NE concentrations under the manufacturer’s instructions. The microplate reader was used to find the OD value at 450 nm (AMR-100, Aosheng, China).

### Histopathological examination of spleen and lung tissues

The lung and spleen tissues were taken out, fixed with 10% formalin, and prepared for histological analysis. The spleen and lung tissues were microtome dissected into 6-m thick sections after being translucent, dehydrated, soaked, and fixed in paraffin wax. The sections were then stained with hematoxylin/eosin (HE) to assess them. A histopathological scoring system (ranging from 0–26) was utilized to assess the pathology of pulmonary infection. We obtained averaging scores from each lung by evaluating the quantity and quality of peri-bronchiolar and peri-bronchial infiltrates, luminal exudates, peri-vascular infiltrates, and parenchyma (Cimolai et al., 1992).

### Assessment of infarct volume

As previously described, the infarct volume was assessed by TTC staining (Liang et al., 2014). Using Image-Pro Plus 6.0 software, the infarct regions and total area on each slice were calculated, and the result was expressed as the percentage of infarction in the entire area.

### Isolation of immune cells from blood, spleen, and brain

Red blood cells (RBCs) were lysed with ACK buffer before being rinsed with PBS. The cells were then maintained on ice until staining. The spleens were minced, and the cell suspension passed through a 40 μm cell strainer, and then cells were treated with ACK buffer to lyse red cells. The homogenates of the brain were centrifuged at 30% Percoll after digestion for half an hour at 37°C in 3 ml of 0.08% trypsin diluted with DMEM, then the cells were gathered as pellets and washed in PBS.

### Flow cytometry

Isolated leukocytes were centrifuged using a 37%–70% Percoll density gradient. After three rounds of washing with buffer (PBS with 0.5% bovine serum albumin and 0.02% sodium azide), cells were stained for 20 min at 4°C with the antibodies listed below. CD3 is a T cell receptor complex that is expressed in T cells. B cells have a cell surface protein called B220 that helps with antigen-based B cell activation. According to CD45 and CD11b expression patterns, we identified three distinct cell populations in the brain: CD11b+CD45low (microglia), CD11bhighCD45high (granulocytes and macrophages), and CD11b−CD45high (lymphoid cells). In the CD11bhighCD45high population, two subpopulations were identified: F4/80 + Gr1+ (activated macrophages) and F4/80−Gr1+(granulocytes). To identify the leukocyte phenotypes, we used FlowJO software for single-cell flow cytometry analysis.

### Quantification of cytokines in cerebral and lung tissues

By using widely available immuno-enzymatic commercial assays specific to equine species, we identified pro and anti-inflammatory cytokines (TNF-α, IL-1β, and INF-γ) (Cloud-Clone Corp, Wuhan, China). Plasma was created by centrifuging whole blood at 1,500 g for 10 min to analyze all cytokines. Following the manufacturer’s instructions, samples were frozen and filtered before being used for analysis using mouse-specific ELISAs (Bioswamp, China).

### Immunofluorescence detection of spleen

For immunofluorescence detection, serial frozen spleen sections were washed in 0.05% BSA in PBS and blocked using species-specific normal serum according to the secondary antibody. Primary antibodies were incubated for 1 h at room temperature. B cells were detected using a monoclonal antibody (mAb) B220 (2.5 μg ml−1) to detect CD45R (BD Pharmingen, United States). T cells were detected using an anti-CD3 antibody (5 μg ml−1) to detect CD3 (2.5 μg ml−1, BD Pharmingen, United States). After adding the primary antibody, sections were washed in TBST buffer (Sigma, United States). And 1 μL Goat Anti-Rat IgG (HRP) secondary antibody (Abbkine, United States) was coupled to Alexa Fluor™ 488, Alexa Fluor™ 555 dyes, or Alexa Fluor™ 647 fluorochromes (Thermo Fisher Scientific, United States) were incubated for 45 min at 37°C. Sections were washed in PBS–BSA and mounted in a fluorescent mounting medium, and images were captured using a Leica Stellaris five confocal microscope (Leica, Wetzlar, German).

Digital images were analyzed using ImageJ software. Three spleens from each experimental group were analyzed. From each spleen, one section, 100 μm apart, was studied, and on each section, we collected data from eight individual areas of white pulp. Fluorescent intensity thresholds were applied, and the number of pixels of each color (green and red) was automatically counted and used to determine the area of immunolabelling for each cell type.

### Statistical analysis

Data are presented as the mean *±* s.d. For statistical analysis, GraphPad Prism software (version 8.0) was employed. The outliers were recognized by the ROUT approach and eliminated from the analysis (Q = 1.000%). A two-tailed Student’s *t*-test was used to compare two groups, a one-way ANOVA with Holm-Sidak correction was used to compare pairs of data from three different groups, and a Mann-Whitney test was used to compare the histopathological score between two groups after verifying that these datasets’ normal distributions are accurate (Kolmogorov-Smirnov test). A *p*-value of less than 0.05 was considered significant, whereas values of less than 0.01, 0.001, and 0.0001 were highly significant.

## Results

### DEX’s effects on NE release

**FIGURE 1:**
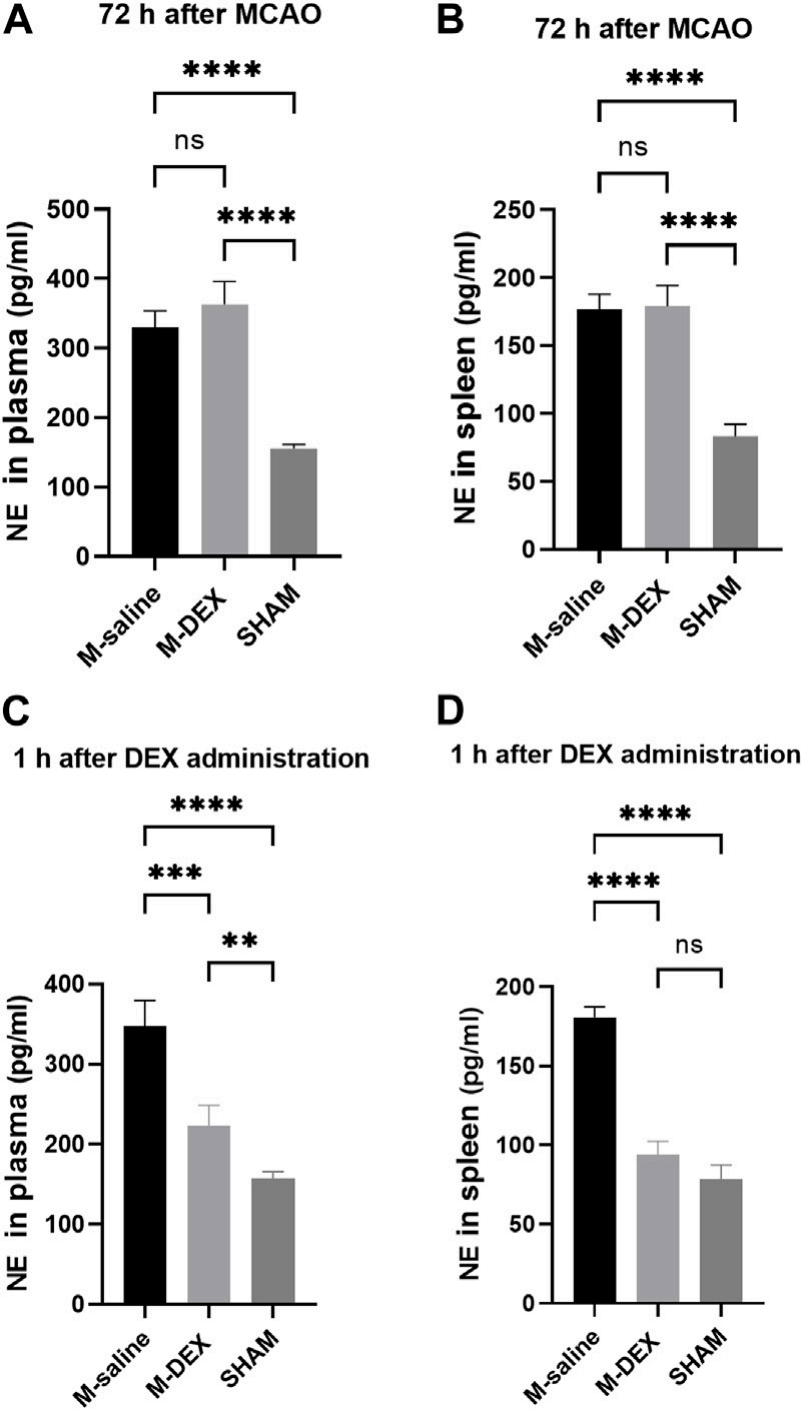
NE concentrations in the mice of the SHAM, M-saline, and M-DEX groups. **(A)** Spleens from the aforementioned three groups were displayed (SHAM *n* = 3, M-saline *n* = 6, M-DEX *n* = 4), and **(B)** spleen index, assessed 72 h after MCAO in the saline and DEX-treated MCAO mice as an index of immunological response (SHAM *n* = 3, M-saline *n* = 6, M-DEX *n* = 4). **(C)** Plasma and **(D)** spleen NE levels in the saline, DEX-treated MCAO mice were assessed 1 h after a stroke (*n* = 4 per group). ** indicates *p* < 0.01, ** indicates *p* < 0.001, and **** indicates *p* < 0.0001 by one-way ANOVA with Holm–Sidak correction.

NE only has anti-inflammatory effects in micromolar quantities (Sander et al., 2001). The spleen’s NE nerve terminals, which produce a significant amount of NE in the vicinity of antigen-activated immune cells, were hypothesized to be primarily responsible for the immune-modulatory impact (Bergquist et al., 1998). We then tested the hypothesis that providing mice with DEX decreases NE levels and shields them from SAP. At first, NE levels in plasma and spleen 72 h after stroke were both assayed, and we observed no reduction in the M-DEX group when compared to the M-saline group (Figures 1A,B). We ascribed the unchanged level of NE at that point to the loss of efficacy caused by the diminished or eliminated concentration of DEX. Hence, we examined the levels of NE in the plasma and spleen 1 h after the three-dose of DEX applications the first day after MCAO. Not surprisingly, NE levels were lower than in the M-saline group, from 352.4 ± 29.1% to 221.7 ± 22.4% in plasma and 176.5 ± 9.8% to 94.1 ± 6.6% in the spleen (Figures 1C,D). The decreasing amplitude reached 37% in plasma and 46.7% in the spleen.

### DEX failed to improve SAP or diminish infarction area 3ds after MCAO

**FIGURE 2:**
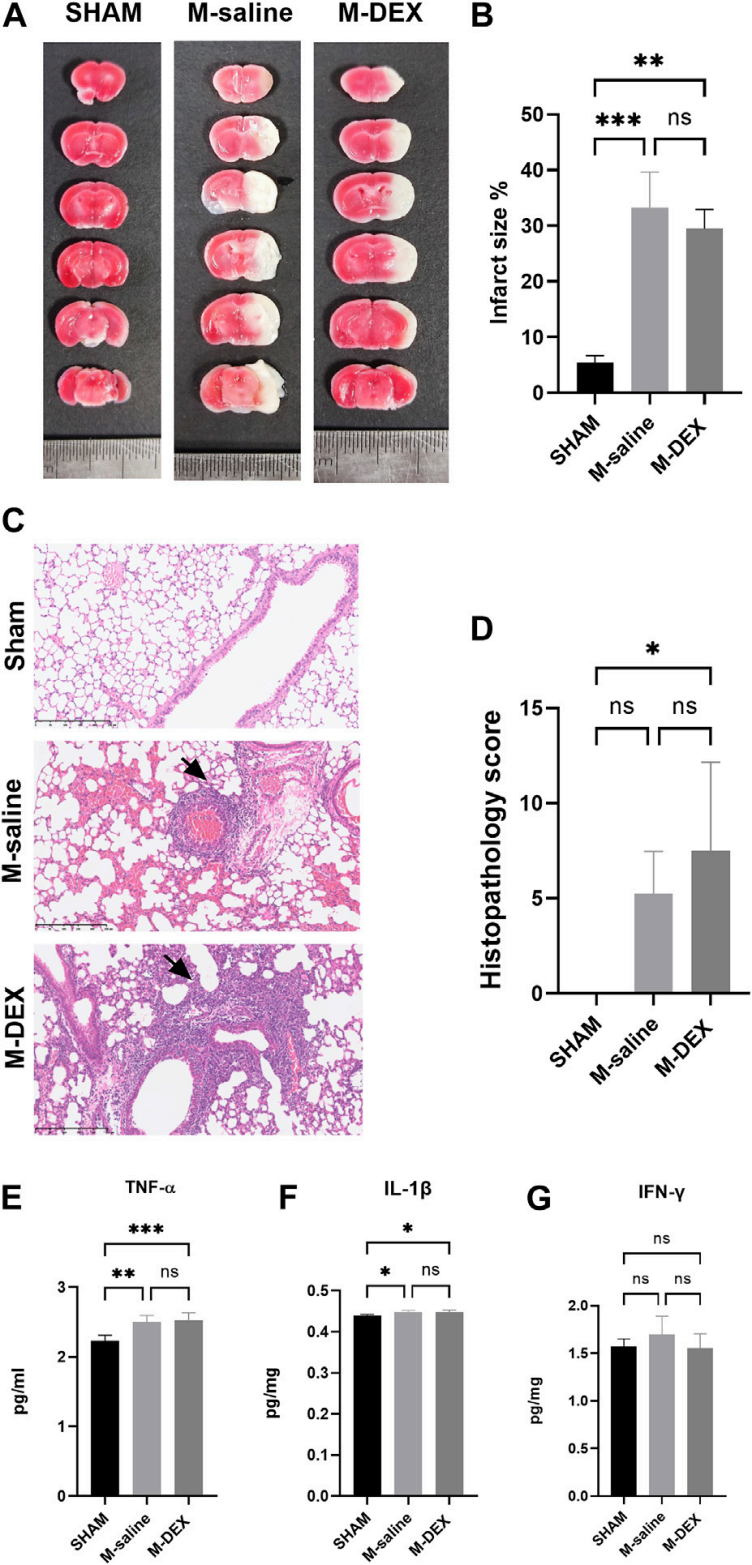
The influence of α2-ARs agonist DEX on stroke-associated pneumonia and infarction area. **(A)** Representative TTC-stained coronal serial sections of cortical infarcts in the saline, and DEX-treated MCAO mice 3 days after stroke. **(B)** Infarct volumes of MCAO in the saline, and DEX-treated MCAO mice 3 days following the stroke (n = 3 per group). **(C)** representative 12-µm section of HE-stained lungs from the SHAM, M-saline, and M-DEX groups, lung sections from saline, and DEX-treated MCAO mice revealed signs of pneumonia (thickening of alveolar walls and neutrophilic infiltrates) ×40. **(D)** Quantitative analysis of histopathological lung injury scores (*n* = 4 animals per group). Effects of DEX on the expression of pro-inflammatory cytokines in the lung, in the groups of SHAM, saline, and DEX-treated MCAO mice 72 h after occlusion (*n* = 5 per group), the levels of pro-inflammatory cytokines **(E)** TNF-α, **(F)** IL-1β, and **(G)** IFN-γ were measured. Mann-Whitney test was used to analyze HPS scores, and one-way ANOVA with Holm–Sidak correction was used for the analysis of cytokines. *, *p* < 0.05, ** indicated *p* < 0.01, and *** indicated *p* < 0.001.

Figure 2A depicts the sites of ischemic lesions 3 days after a stroke. The average size of the ischemic lesions in the stroke mice spanned 33.3% of the entire brain, and there were no changes between the M-DEX group’s infarct volume and that of the MCAO group, which was 29.5 ± 3.5% (Figure 2B).

Images of the HE-stained lung sections from the three groups, including the SHAM, M-saline, and M-DEX groups, are shown in Figure 2C. Inflammatory pathological changes including thickened alveolar septa, intra-alveolar inflammatory infiltration, and interstitial congestion were seen in the sections of the M-saline and M-DEX groups. Figure 2D displays the histopathological results for the three groups (the Sham, M-saline, and M-DEX groups). The findings demonstrated that all the mice developed pneumonia 72 h after an MCAO stroke, and the HPS in the M-saline group were all below ten, while two in four of the HPS in the M-DEX group were above ten. When TNF-α, IL-1β, and IFN-γ were analyzed as pro-inflammatory cytokines, we found that DEX therapy did not affect their expression in the lung (Figures 2E–G). Combing these two findings, we concluded that DEX treatment provided no reduction or alleviation of pneumonia, DEX-treated MCAO mice may even have a higher susceptibility to pneumonia or more severe inflammatory sign of pneumonia than the saline-treated MCAO mice.

### Effects of DEX administration on the spleen, including spleen index, and its morphological and immunological alterations

**FIGURE 3:**
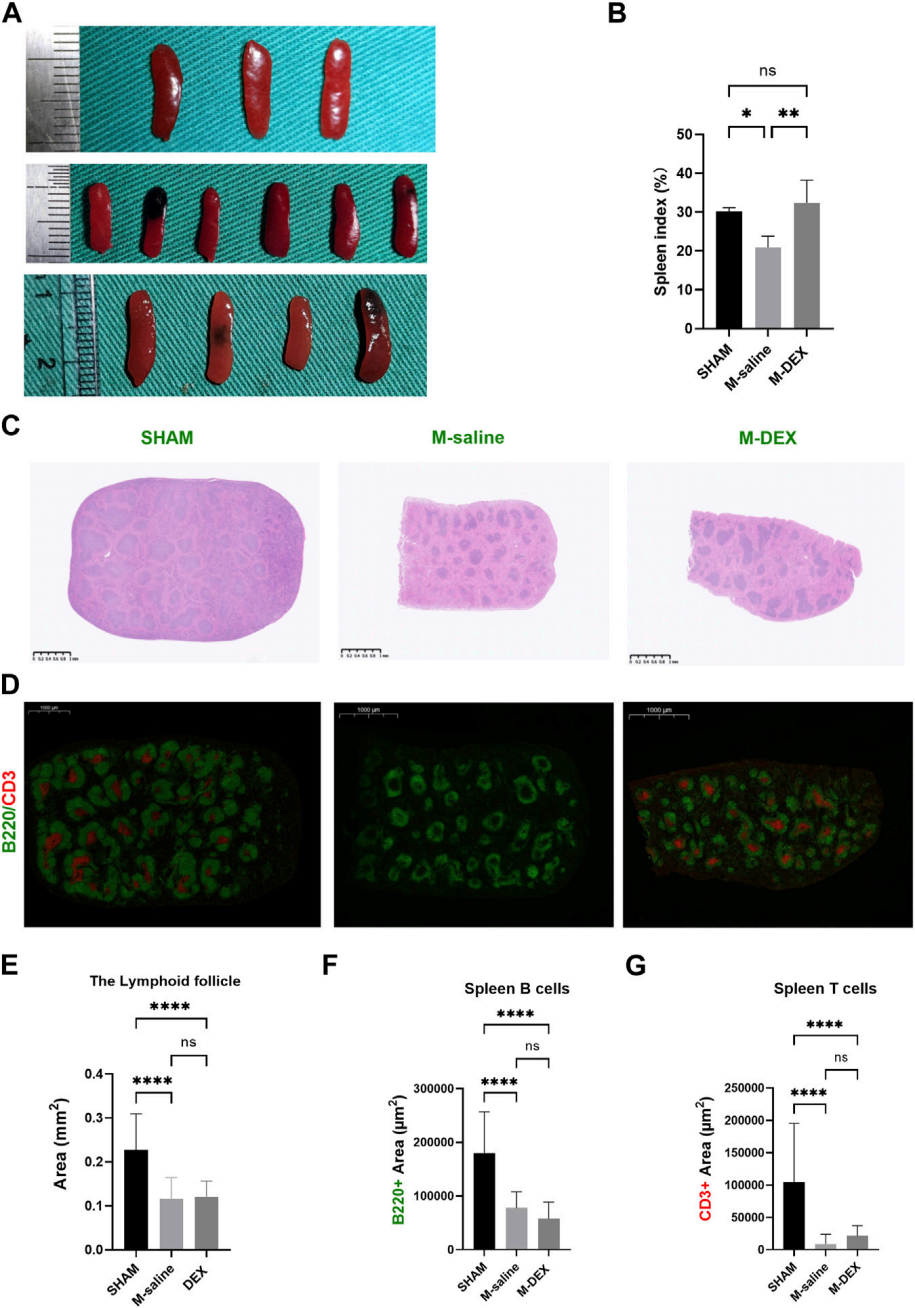
Effects of DEX on the spleen, including spleen index, and alterations to its morphology and immunology. **(A)** Histological sections of spleens in the SHAM, M-saline, and M-DEX groups. **(B)** Spleen index in the SHAM, M-saline, and M-DEX groups (SHAM *n* = 3, M-saline *n* = 6, M-DEX *n* = 4) **(C)** showed histological sections of spleens in the SHAM, M-saline, and M-DEX groups, and multiple B cell follicles can be seen. **(D)** displays fluorescent histological sections of spleens in the SHAM, M-saline, and M-DEX groups, B cells were immunolabelled with B220 antibody (green), and T cells with CD3 antibody (red). **(E)** Quantification of the areas of lymphoid follicles in the three groups 3 days after MCAO (*n* = 3 per group). **(F)** The absolute measurement of B220 fluorescence, and **(G)** CD3^+^ fluorescence areas, as a measure of B cells and T cells respectively (*n* = 3 per group). Data were shown as mean ± s.d. *p < 0.05, **p < 0.01; ***p < 0.001; ****p < 0.0001, one-way ANOVA with Holm–Sidak correction was used to compare each column to every other column among the three groups.

As shown in Figure 3A, we noticed that the spleen dramatically shrank at 3ds after MCAO, and DEX administration significantly increased the spleen size that MCAO induced. The administration of DEX also countered the reduction of the spleen weight in MCAO mice (data was not shown). The results showed that the spleen index in the M-DEX group was significantly higher than in the M-saline group, indicating that DEX could restore the peripheral immunity that had been weakened by MCAO (Figure 3B).

The morphological features in the spleen were also disrupted by MCAO, mainly manifested as the shrank size of the B-cell follicle after MCAO, accompanied by a decrease in the areas of B220 fluorescence, suggesting a reduction in the B-cell population in the spleen (Figures 3C,E,F). In our results, the CD3^+^ fluorescence vanished in two of the three spleens in the MCAO group, while all the CD3^+^ fluorescence in the M-DEX group though diminished remarkably, remained, indicating that there was a sharp drop in the T cell population after the MCAO procedure (Figure 3D). The mean CD3^+^ fluorescence measures in the M-DEX group were higher than those of the M-saline group, suggesting that CD3^+^ T cells in the spleen had partially recovered following DEX treatment (Figure 3G). However, the B220 fluorescence region had no differences between the two groups (Figure 3F).

### Effects of DEX therapy on the ratio of lymphocyte, and the two major lymphocyte subtypes, T and B cells, in the spleen, blood, and brain

**FIGURE 4:**
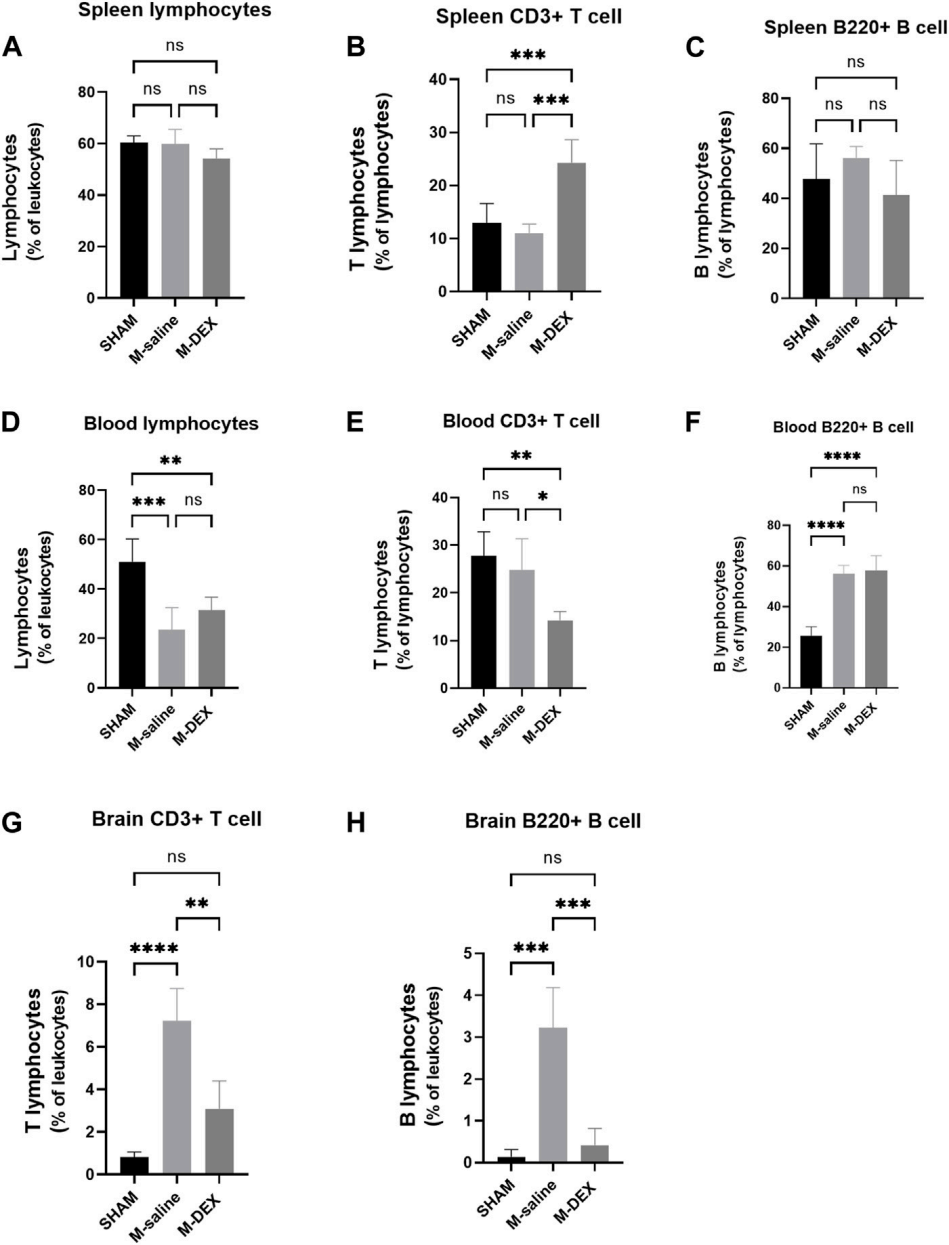
Effects of DEX on the ratios of total lymphocytes, as well as the two main subsets of lymphocytes T-cells and B-cells, in the spleen, blood, and brain. Cell subpopulation percentages in the SHAM, M-saline, and M-DEX groups 72 h after stroke, including **(A)** total lymphocytes, **(B)** total CD3^+^ T cells, and **(C)** B cells (B220+) in the spleen (n = 5 per group); **(D)** total lymphocytes, **(E)** total CD3^+^ T cells, and **(F)** B cells (B220+) in the blood (n = 5 per group); **(G)** total CD3^+^ T cells and **(H)** B cells (B220+) in the brain (n = 5 per group). * signifies *p* < 0.05, **p < 0.01, ***p < 0.001, and ****p < 0.0001.

We questioned whether there was a connection in the populational alteration of the T and B cell population among the spleen, blood, and brain since DEX influenced the splenic T and B cell populations and the lymphocyte population in the ischemia brain. The proportion of lymphocytes, T, and B cells in the spleen was additionally examined by flow cytometry at first. The findings indicated that there was a general decrease in the total cell population in the spleen after MCAO stroke because none of the three types of proportions altered between the SHAM and M-saline groups (Figures 4A–C). The proportion of CD3^+^ T cells significantly increased after DEX treatment, and this result was consistent with the immunofluorescence of the spleen results, which showed a reserved red fluorescence in all the MCAO spleen, indicating that DEX helped to maintain more T cells in the spleen after MCAO.

Next, the peripheral blood was analyzed to determine the percentage of T, B, and total lymphocytes. The M-saline group in our study had a much-decreased blood lymphocyte population, as seen in other studies, which suggested that the MCAO mice experienced immunological suppression (Figure 4D). However, compared to the SHAM group, our research showed a greater B cell population in the M-saline and M-DEX groups (Figure 4F), the elevation of the B cell population in the two groups may result from the abundant expulsion of B cells from the spleen. Both of the above changes in the stroke mice were not affected by DEX treatment. No variation of the T cell population in the MCAO groups compared to the SHAM group may be due to less expulsion of T cells from the spleen, but higher composition of T cell population in the blood. Our research revealed that the T cell population in the blood reduced after DEX treatment, which contrasted with the finding that more T cells were retained in the spleen after DEX treatment (Figure 4E).

Since the ischemic brain is the primary site that induces immune changes in the whole body, we examined its effects on the ratio of T cells to B cells in the ischemic brain and discovered that both ratios were enhanced, with 7.23% of T cell populations and 3.23% of B cell populations. The amplitude of rise in the T cell population was noticeably greater than that in the B cell population. Following DEX therapy, both of the two populations drastically decreased (Figures 4G, H).

### DEX’s effects on the primary cerebral immune state in the stroke mice

**FIGURE 5:**
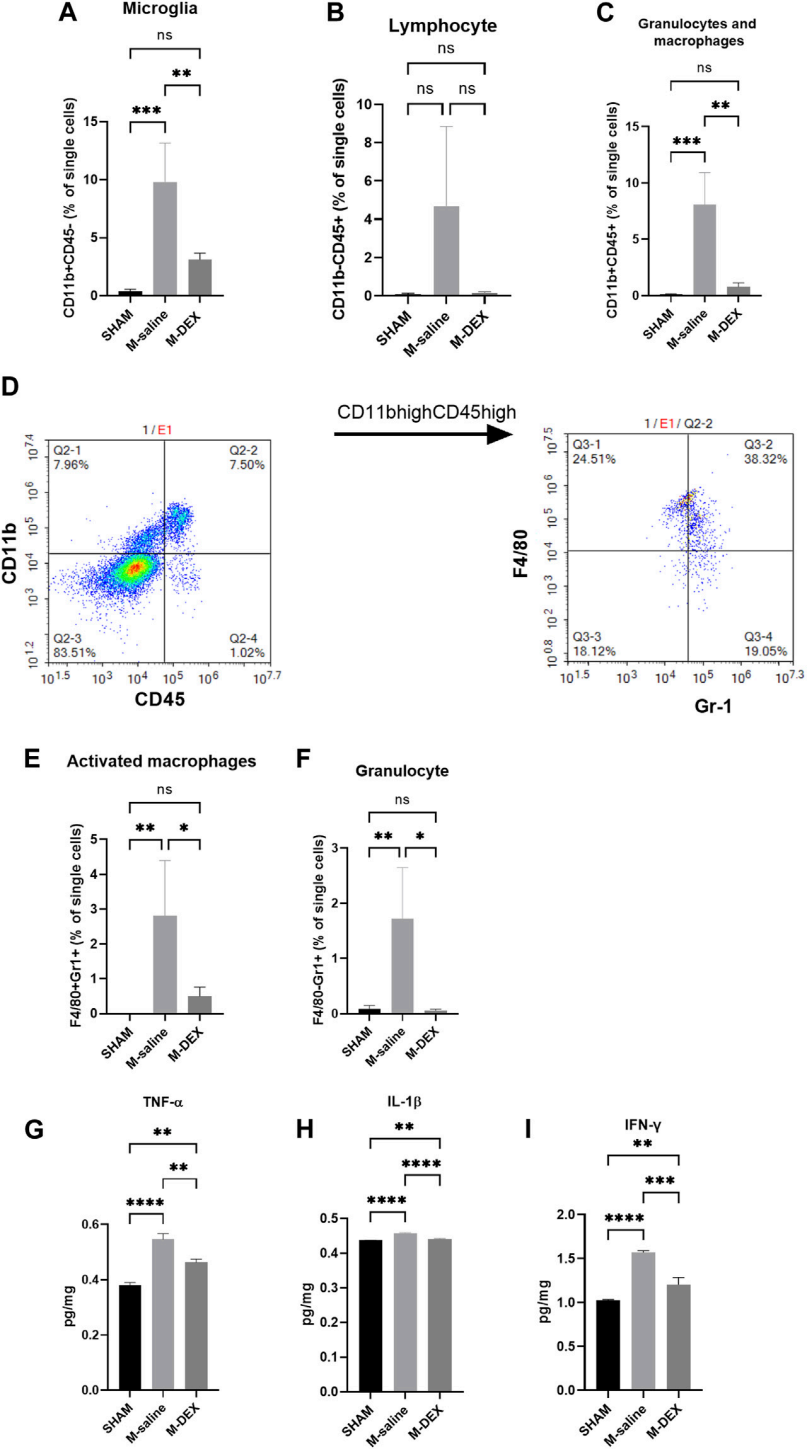
Effects of DEX on central immunity. Percentages of immune cell subpopulations including **(A)** microglia, **(B)** lymphocytes, **(C)** granulocytes, and macrophages in the cerebral 72 h after stroke in the SHAM, M-saline, and M-DEX groups (SHAM *n* = 4, M-saline *n* = 4, M-DEX *n* = 3) were assessed. Then, granulocytes and macrophages were sorted further with CD11b and CD45 **(D)**. **(E, F)** show granulocytes and macrophages, respectively. We also analyzed the effects of stroke and DEX application on the expression of pro-inflammatory cytokines in the cerebral brain in MCAO models. Pro-inflammatory cytokines including **(G)** TNF-α, **(H)** IFN-γ, and **(I)** IL-1β levels in sham-operated and saline, DEX-treated MCAO mice 72 h after occlusion. (One-way ANOVA with Holm-Sidak correction, *n* = 3 per group in all the analyses of cytokines) *, *p* < 0.05, **, *p* < 0.01, ***, *p* < 0.001, and ****, *p* < 0.0001.

We then tested the main immune cell populations, identified with CD11b and CD45 in the ischemic brain. We found that all the immune cell populations, including microglial cells, lymphocytes, granulocytes, and macrophages, increased after MCAO. The magnitudes of change in lymphocyte, granulocyte, and macrophage populations were more obvious than in the microglial population (Figures 5A–C). DEX treatment largely reversed the impact of ischemia on the immune cell populations (Figures 5A–C). The population of granulocyte and macrophage was further analyzed with Gr1 and F4/80 markers to identify each separately. In the CD11bhighCD45high population, two subpopulations were identified (Figure 5D): F4/80 + Gr1+ (activated macrophages) and F4/80−Gr1+ (granulocytes). There were more activated macrophages and significantly higher numbers of granulocytes in the M-saline group than in the SHAM group, and DEX inhibited the increase (Figures 5E,F).

The impact of DEX on the immuno-state of the stroked cerebral ischemic brain was unclear. First, we looked at cytokine generation in the ischemic brain. In the MCAO mice, levels of the pro-inflammatory cytokines TNF-α, IL-1β, and IFN-γ were all increased (Figures 5G–I). When DEX was administered to MCAO mice, the production of pro-inflammatory cytokines was largely reversed.

### DEX delivery caused enlarged spleen as well as pneumonia in the normal mice

Spleen images from the sham mice treated with PBS and DEX are individually shown in Supplement Figure A. It was obvious to see that the spleens in the DEX-treated sham mice were digested with blood, and larger than the PBS-treated ones, and the spleen index rose from 21.7% to 42.7% in the DEX-treated sham mice (Supplement Figure B). We discovered that 72 h after the MCAO procedure, all sham mice treated with DEX developed pneumonia, with substantial infiltration of inflammatory cells. These findings demonstrated that DEX treatment after MCAO stroke will not prevent mice from developing pneumonia, but would rather make their lungs worse.

## Discussion

This study assessed the impact of DEX, a selective α2-AR agonist, on the central and peripheral immune system, as well as its effects on cerebral and lung tissues, given the fact that α2-ARs play a crucial role in down-regulating the SNS activity (Yuan et al., 2020), the enhancement of which is regarded as the underlying cause of SAP. In contrast to the untreated MCAO animals, DEX resulted in a lower blood T cell population but a higher retained T cell population in the spleen. However, SAP was not improved.

Stroke mice displayed a stronger sympathetic tone than sham mice, as shown by higher NE levels (Xu et al., 2022). It is well-recognized that the depressed immune state that was brought about by increased sympathetic tone was the main cause of SAP (Faura, et al., 2021). Massive sympathetic discharge triggers lung inflammation (Avlonitis et al., 2005). In addition, providing animals with NE can also result in pulmonary edema and inflammation (Rassler et al., 2003). Thus, we hypothesized that lowering NE levels in MCAO mice might alleviate lung inflammation. It is common knowledge that DEX would reduce NE levels (Nelson et al., 2003; Kang et al., 2020). We first analyzed the plasma and the spleen NE levels, when samples were collected 24 h after the last dose of DEX, that is, 72 h after stroke, and no variation was observed between M-saline and M-DEX group. We then analyzed the NE levels again with samples collected 1 h after the three doses of first-day DEX application and verified that both the plasma and spleen NE levels were decreased as supposed. Moreover, we noticed that DEX treatment enlarged MCAO mice’s shrunk spleen, a characteristic associated with severe stroke (Seifert et al., 2012). Since NE has been demonstrated to cause significant splenic atrophy (Mignini et al., 2003), the increase in spleen size may be explained by the decreased NE levels brought by DEX.

Given that the spleen is a reliable indicator of immune function, and the sizes varied between the three groups, we measured the morphological and immunological alteration of the spleen in the MCAO mice to ascertain whether DEX administration would reverse the intrinsic effects that were caused by MCAO. As the largest secondary lymphoid organ, the spleen serves as the reservoir mainly for two subsets of lymphocytes, B- and T-lymphocytes, and plays a crucial role in initiating the immune response. The B220+ B-cell follicles in the MCAO spleen showed a shrinkage, which was consistent with the change of the follicles presented by HE images. Inconsistent with the changes in the spleen size, DEX did not increase the size of the B-cell follicles. As to the CD3^+^ T-cells, their aggregation disappeared in the MCAO spleen, while DEX treatment partially restored the aggregation of the CD3^+^ T-cells. The flow cytometry analysis showed that the ratio of total lymphocytes, and the two subsets, B- and T-lymphocytes were not altered in the MCAO spleen when compared to the Sham group, indicating that there was an overall decline of cells in the spleen. In line with immunofluorescence’s findings, DEX treatment raised the ratio of CD3^+^ T-cells in the spleen while maintaining the ratio of total lymphocytes to B-lymphocytes.

Emerging research indicates that ischemic stroke evokes spleen contraction and disrupts the peripheral immune system. As a result, we looked at the proportions of total lymphocytes and the two major subgroups, B- and T-lymphocytes, in the peripheral blood. As demonstrated in prior research, we also established that MCAO stroke caused a significant decline in the percentage of total lymphocytes. However, the ratio of the B- and T-lymphocytes to the total lymphocytes were not altered, indicating that there was a total loss of B- and T-lymphocytes in the blood after the MCAO stroke. DEX application made no change in the proportion of the total lymphocytes but decreased the ratio of the CD3^+^ T-cells to the total lymphocytes. We postulated that DEX may depress immunity by preventing the recruitment of CD3^+^ T-cells from the spleen to the blood because the proportions of the blood CD3^+^ T-cells were decreased and the spleen CD3^+^ T-cells were increased after DEX administration.

As the origin of the immune responses, the immuno-state of the ischemic brain was further examined. First, the ratios of the total lymphocytes and the two subsets, B- and T-lymphocytes, were studied. The results demonstrated that overall lymphocyte ratios, as well as ratios of B- and T-lymphocytes, increased after MCAO stroke, with the rise in CD3^+^ T-cells being more prominent. Treatment with DEX could inhibit all ratio increases mentioned above. Then, we looked at the changes in the proportion of other immune cells and the expression of pro-inflammatory cytokines. We discovered that the number of residual microglia was unaffected by MCAO, even though that activated microglia were reported to be the predominant phagocytic cells in the ischemic brain (Schilling et al., 2005). We hypothesized that this was because microglial cells were more activated than proliferated. However, 3 days after MCAO, the blood-born population of activated macrophage and granulocyte cells all showed dramatic increases, and DEX therapy led to a widespread, significant decrease of immune cell populations in the ischemic brain. Accordingly, cytokine analysis showed that pro-inflammatory cytokines, including TNF-α, IL-1β, and IFN-γ, were up-regulated in the MCAO brain, and DEX treatment inhibited the expression of these pro-inflammatory cytokines. The suppression of DEX on immunological responses in the brain suggested that fewer peripheral immune cells would be recruited by the ischemic brain.

As shown by our study, DEX’s anti-inflammatory effects on the ischemic brain did not aid to diminish the infarction area. It had been established that inhibition of the neuroinflammatory response following ischemic stroke would even increase stroke size (Lechtenberg et al., 2019). Considering DEX’s ability to lower peripheral sympathetic tone, we reasoned that its administration would boost peripheral immunological response and lessen the MCAO mice’s susceptibility to pneumonia. However, all mice developed pneumonia after DEX treatment, and HPS scores were equal to or higher than those of the mice in the M-saline group. The immune suppression induced by stroke is mainly characterized by a reduced lymphocyte population. DEX’s application to the MCAO mice didn’t help to improve the lymphocyte ratio, even worse, caused a decrease in the CD3^+^ T-cell population.

The decrease in total T-cells denotes an inhibited immunological performance. Previous studies implied that immunodepression, characterized by total T-cell reduction was the cause of SAP (Faura et al., 2021). Plentiful studies have shown that elevations in NE or activation of β2-ARs can induce CD3^+^ T cell lymphopenia (Huang et al., 2010; Oros-Pantoja et al., 2011; Zieziulewicz et al., 2013). Even though DEX decreased NE levels, it also caused a decline in the blood CD3^+^ T cells. Furthermore, it is commonly recognized that NE production and subsequent activation of the β2-ARs are the causes of SAP and that blocking *β*-ARs with propranolol can help avoid SAP while DEX administration has no therapeutic effects on SAP. In conclusion, we considered that DEX’s impact on the immune cell distribution was irrelevant to the decline of NE concentration. As stated above, DEX had been proved to reduce sepsis induced lung inflammation through a variety of anti-inflammatory pathways. Different theories of pneumonia, in our opinion, account for the variations in DEX’s effects. For instance, peripheral inflammation characterizes pneumonia brought on by LPS, and DEX’s anti-inflammatory effect is helpful in reducing the inflammatory response. However, peripheral immunosuppression characterizes pneumonia brought on by MCAO mice, and DEX’s anti-inflammatory effect worsens the condition, which is not helpful for SAP prognosis.

We also looked at how DEX might affect the pulmonary tissues of normal mice and how the size of their spleens changed after being treated with DEX. We administered DEX to the normal mice with the same dose and procedure as we did to the MCAO mice, and found that after exposure to DEX, all mice developed pneumonia. Additionally, larger spleens were observed in the DEX-treated group than in the PBS-treated group, suggesting an alteration of immunity occurred after DEX application. DEX is widely accepted as an anti-inflammatory drug, which has been shown to suppress inflammatory responses in multiple organs (Bao and Tang, 2020). Although the decrease in spleen size matched that of the MCAO mice, it was unclear what specifically had happened to the distribution of immune cells in the normal mice treated with DEX. Hence, it remains to be determined whether the decrease in CD3^+^ T cells was the cause of DEX’s involvement in the lung in both MCAO and normal mice.

### Limits

There are still some limitations in our study. First, it is necessary to clarify the specific immunological changes in DEX-treated normal mice and compare them to those in DEX-treated MCAO mice, which may shed light on whether SAP is caused by a decrease in the blood CD3^+^ T cells; Second, the mechanism by which DEX caused the decline in the blood CD3^+^ T cells has not been figured out. Hence, further research is required.

## Conclusion

This work emphasizes the impact of DEX on post-stroke central and peripheral immunological responses and outcomes, particularly the implications of SAP. According to our research, we believe DEX may not be appropriate for stroke patients. The reasons are outlined below. First, DEX produced a fall in the blood CD3^+^ T cells, which may exacerbate immunological depression in SAP mice; Second, combining the effects of DEX on the histology of the lungs in SAP mice or normal mice, DEX medication may induce lung injury.

## Data Availability

The original contributions presented in the study are included in the article/supplementary material, further inquiries can be directed to the corresponding authors.
